# Glycan Masking of Hemagglutinin for Adenovirus Vector and Recombinant Protein Immunizations Elicits Broadly Neutralizing Antibodies against H5N1 Avian Influenza Viruses

**DOI:** 10.1371/journal.pone.0092822

**Published:** 2014-03-26

**Authors:** Shih-Chang Lin, Wen-Chun Liu, Jia-Tsrong Jan, Suh-Chin Wu

**Affiliations:** 1 Institute of Biotechnology, National Tsing Hua University, Hsinchu, Taiwan; 2 Genomics Research Center, Academia Sinica, Taipei, Taiwan; 3 Department of Medical Science, National Tsing Hua University, Hsinchu, Taiwan; Georgia State University, United States of America

## Abstract

The highly pathogenic avian influenza (HPAI) H5N1 virus, a known trigger of diseases in poultry and humans, is perceived as a serious threat to public health. There is a clear need for a broadly protective H5N1 vaccine or vaccines for inducing neutralizing antibodies against multiple clades/subclades. We constructed single, double, and triple mutants of glycan-masked hemagglutiinin (HA) antigens at residues 83, 127 and 138 (i.e., g83, g127, g138, g83+g127, g127+g138, g83+g138 and g83+g127+g138), and then obtained their corresponding HA-expressing adenovirus vectors and recombinant HA proteins using a prime-boost immunization strategy. Our results indicate that the glycan-masked g127+g138 double mutant induced more potent HA-inhibition, virus neutralization antibodies, cross-clade protection against heterologous H5N1 clades, correlated with the enhanced bindings to the receptor binding sites and the highly conserved stem region of HA. The immune refocusing stem-specific antibodies elicited by the glycan-masked H5HA g127+g138 and g83+g127+g138 mutants overlapped with broadly neutralizing epitopes of the CR6261 monoclonal antibody that neutralizes most group 1 subtypes. These findings may provide useful information in the development of a broadly protective H5N1 influenza vaccine.

## Introduction

The highly pathogenic avian influenza (HPAI) H5N1 virus, a known trigger of diseases in poultry and humans, is perceived as a serious threat to public health. Two outbreaks occurred in 1997 and 2003; between 2003 and the end of December 2013, the World Health Organization (WHO) received reports of 648 laboratory-confirmed human cases with a mortality rate of approximately 60% [1]. The continuing evolution of H5N1 viruses is raising concerns about a potential human pandemic due to their bird-to-human transmission capability. Researchers have also reported that several mutations in HA and PB2 proteins support H5N1 transmission among ferrets [2], [3]. Reassortant H5N1 viruses bearing 2009/H1N1 virus genes have also been identified in guinea pigs [4], suggesting that HPAI H5N1 viruses are capable of adapting so as to support transmission in other mammals. Novel H7N9 viruses showing Q226L or Q226I mutations in HA associated with mammalian adaptation indicate potential for preferential binding to α-2,6-linked sialic acids for effective human-to-human transmission [5], [6]. H5N1 viruses have been classified into 10 clades, with recently isolated viruses classified into additional subclades based on phylogenetic analyses of viral hemagglutinin (HA) sequences [7].

The WHO is following a vaccine development strategy of creating candidate vaccines as new viruses emerge, resulting in the current list of 27 potential vaccines in response to 12 clades/subclades. There is a clear need for a broadly protective H5N1 vaccine or vaccines for inducing neutralizing antibodies. Arguably the most noteworthy attempts involve the use of AS03 [8], MF59 [9], and the immune stimulating complex adjuvant Matrix M [10]. Other cross-protection strategies include the use of inactivated virus vaccines containing multi-clade [11], [12] or ancestral H5N1 virus strains [13]. DNA vaccines for inducing cross-clade neutralizing antibodies associated with multi-clade HA or consensus HA gene(s) are also in various stages of development [14]–[18].

We previously reported that N-linked glycan masking in highly variable sequences in the HA1 globular head in residues 83 and 127 resulted in increased cross-neutralizing antibody titers [19]. Our goal in this study is to use adenovirus vector prime and recombinant HA protein booster regimens to further investigate cross-clade immunity elicited by single or multiple glycan-masked HAs. Our results indicate that multiple glycan-masked HA elicited the highest titer of cross-clade hemagglutination inhibition (HI) and neutralizing antibodies with enhanced binding to receptor binding sites (RBS) and the stem region. We believe our findings provide useful data in support of the development of broadly protective H5N1 influenza vaccines.

## Results

### Glycan-masked H5HA at Residues 83, 127 and 138

**Figure 1:**
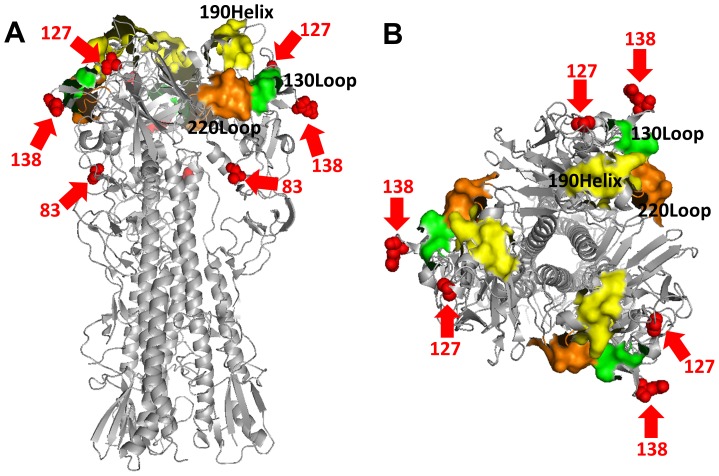
A three-dimensional model of the KAN-1 HA structure. The structure was generated by SWISS-MODEL based on the crystal structure of H5HA (A/Vietnam/1194/04, PDB ID: 2IBX). Images were created with PyMOL 1.3. RBS is composed of 130 loop, 190 helix, and 220 loop. Arrows indicate residues 83, 127 and 138.

**Figure 2:**
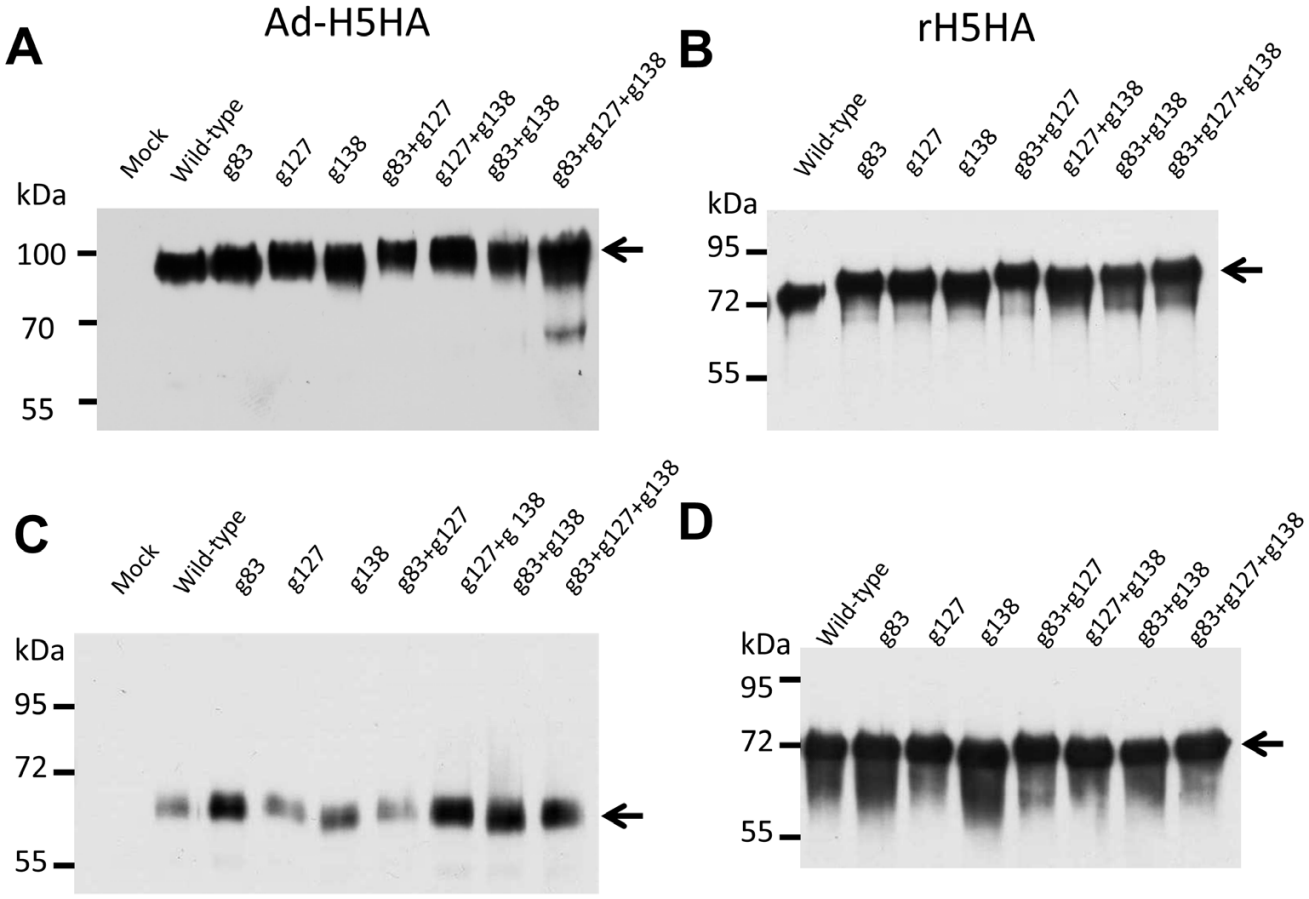
Expression of glycan-masked H5HA mutants. Single, double, and triple mutants of glycan-masked H5HA antigens at residues 83, 127 and 138 were constructed, and their corresponding HA-expressing adenovirus vectors and recombinant HA proteins were obtained. The increased molecular weights of (A) adenovirus-expressed H5HA mutants and (B) Sf9-expressed recombinant H5HA proteins were confirmed by Western blotting. Deglycosylated forms of (C) adenovirus-expressed H5HA mutants and (D) Sf9-expressed H5HA mutants were also confirmed following treatment with PNGase F.

We previously reported that glycan-masked H5HA at residues 83, 127, and 138 did not affect red blood cell agglutination, but only the g83 and g127 mutants induce more potent and broader neutralizing antibodies against H5N1 viruses [19]. In this study, the glycan-masked g138 mutant, which mutated to ^138^NGT^140^ (data not shown) instead of ^138^NRT^140^ used in the previous report [19], was able to induce broadly neutralizing antibodies similar to the glycan-masked g83 and g127 mutants. As elucidated in the three-dimensional H5HA structures shown in Figure 1, residues 127 and 138 are located on the outer HA surface, close to the 130 loop of the receptor binding site (RBS). Residue 83 is located near the HA monomer interface that is observable from a side view (Figure 1A) but not from a top view (Figure 1B). For the present study we constructed single, double, and triple mutants of glycan-masked H5HA antigens at residues 83, 127 and 138 (i.e., g83, g127, g138, g83+g127, g127+g138, g83+g138 and g83+g127+g138), and then obtained their corresponding HA-expressing adenovirus vectors and recombinant HA proteins. These mutants were found to have increased molecular weights for both H5HA protein adenovirus vectors (Figure 2A) and recombinant H5HA proteins (Figure 2B) compared to the wild type H5HA constructs. However, molecular weights were equal following PNGase F treatment (Figure 2C, D).

### H5HA-specific Total IgG Titers Elicited by Glycan-masked H5HA Mutant Antigens

**Figure 3:**
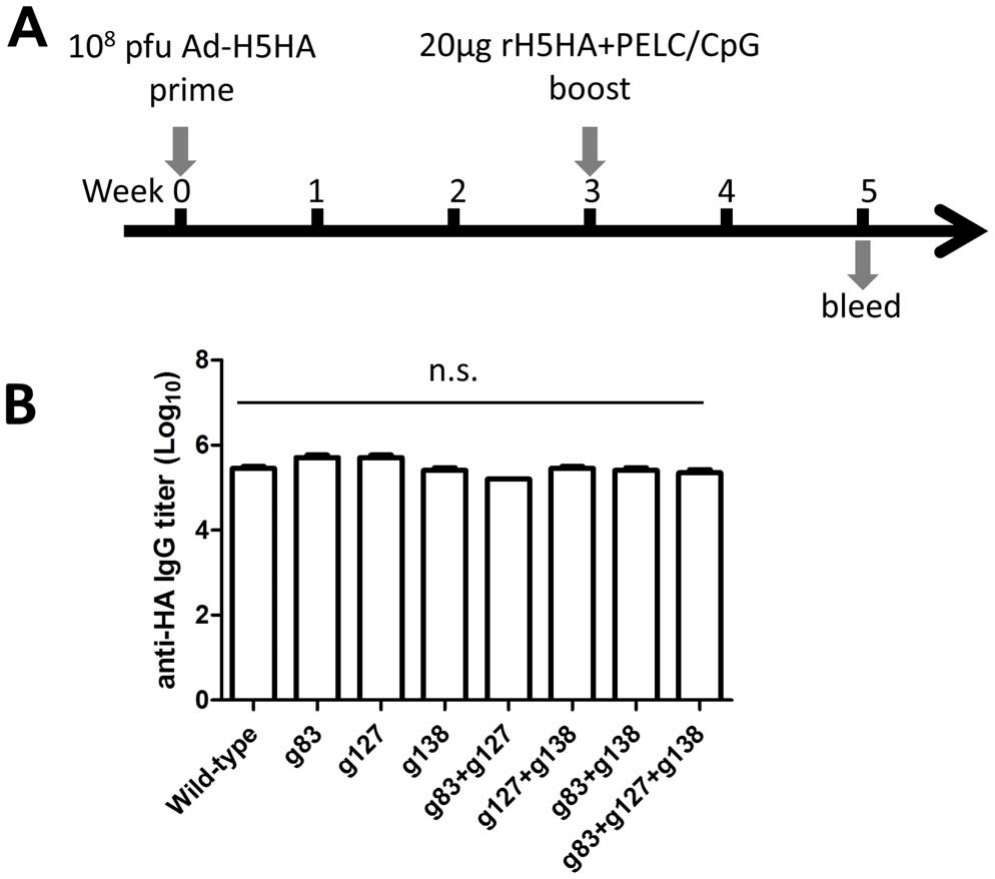
Glycan-masked H5HA immunizations. (A) Immunization regimen by adenovirus prime and recombinant protein booster (B) H5HA-specific IgG titers elicited by the individual glycan-masked H5HA immunizations were determined by ELISAs. Data represent geometric mean ± standard deviation; one-way ANOVA and Tukey’s test results indicate no significant (n.s.) differences.

We previously reported that a heterologous prime-booster immunization regimen using an adenovirus vector and recombinant HA protein elicits more potent neutralizing antibodies against homologous and heterologous H5N1 virus clades [20]. To evaluate the heterologous neutralizing antibody responses elicited by glycan-masked mutant antigens, groups of 6- to 8-week-old female BALB/c mice were primed with 10^8^ pfu of H5HA-encoding adenovirus vector followed by a booster of 20 μg recombinant H5HA proteins coupled with PELC/CpG 3 weeks later [20], [21] (Figure 3A). According to analyses of serum samples collected two weeks following the booster doses, no significant differences were noted in the H5HA-specific IgG titers elicited by each type of glycan-masked H5HAs compared to those elicited by the wild-type immunizations (Figure 3B).

### HI and Neutralizing Antibody Titers Elicited by Glycan-masked H5HA Mutant Antigens

**Figure 4:**
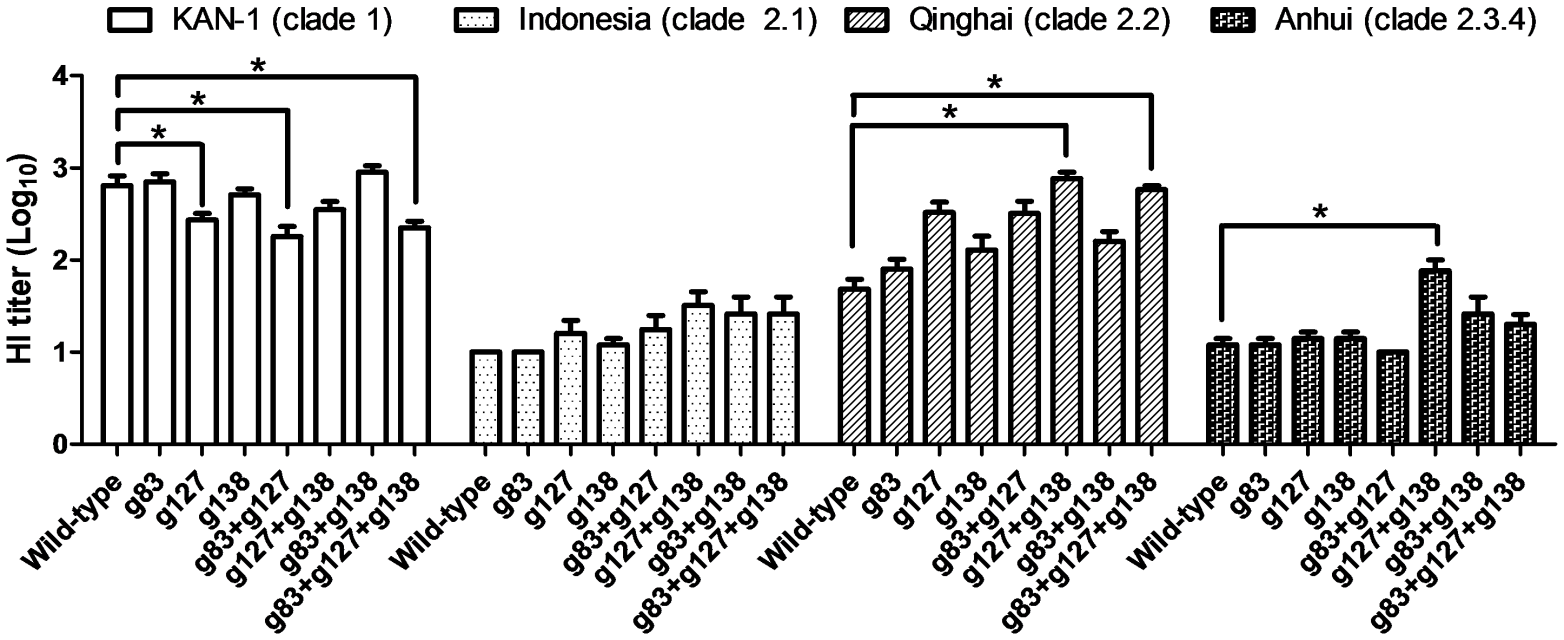
HI titers elicited by glycan-masked H5HA mutant antigens. Sera were serially diluted and incubated with 4 HA units H5N1pp containing HA from KAN-1 (clade 1), Indonesia (clade 2.1), Qinghai (clade 2.2), or Anhui (clade 2.3.4). HI titer was measured as the reciprocal of the highest dilution of sera which completely inhibiting hemagglutination. Data represent geometric mean ± standard deviation. Results were analyzed using one-way ANOVAs and Tukey’s tests (*, statistical significance at p<0.05).

All of the glycan-masked mutants except for g127, g83+g127, and g83+g127+g138 retained similar levels of HI titers against homologous H5N1 (KAN-1, clade 1)-pseudotyped particles (H5pp) (Figure 4). For HI titers against three heterologous forms of H5pp (Indonesia, clade 2.1; Qinghai, clade 2.2; Anhui, clade 2.3.4), all of the glycan-masked mutants except for g83 elicited slightly higher HI titers for the Indonesia clade 2.1 H5pp. Titers elicited by the glycan-masked gp127+g138 and g83+g127+g138 mutants were significantly higher for the Qinghai clade 2.2 H5pp, and titers for the glycan-masked g127+g138 mutant were significantly higher for the Anhui clade 2.3.4 H5pp (Figure 4).

**Figure 5:**
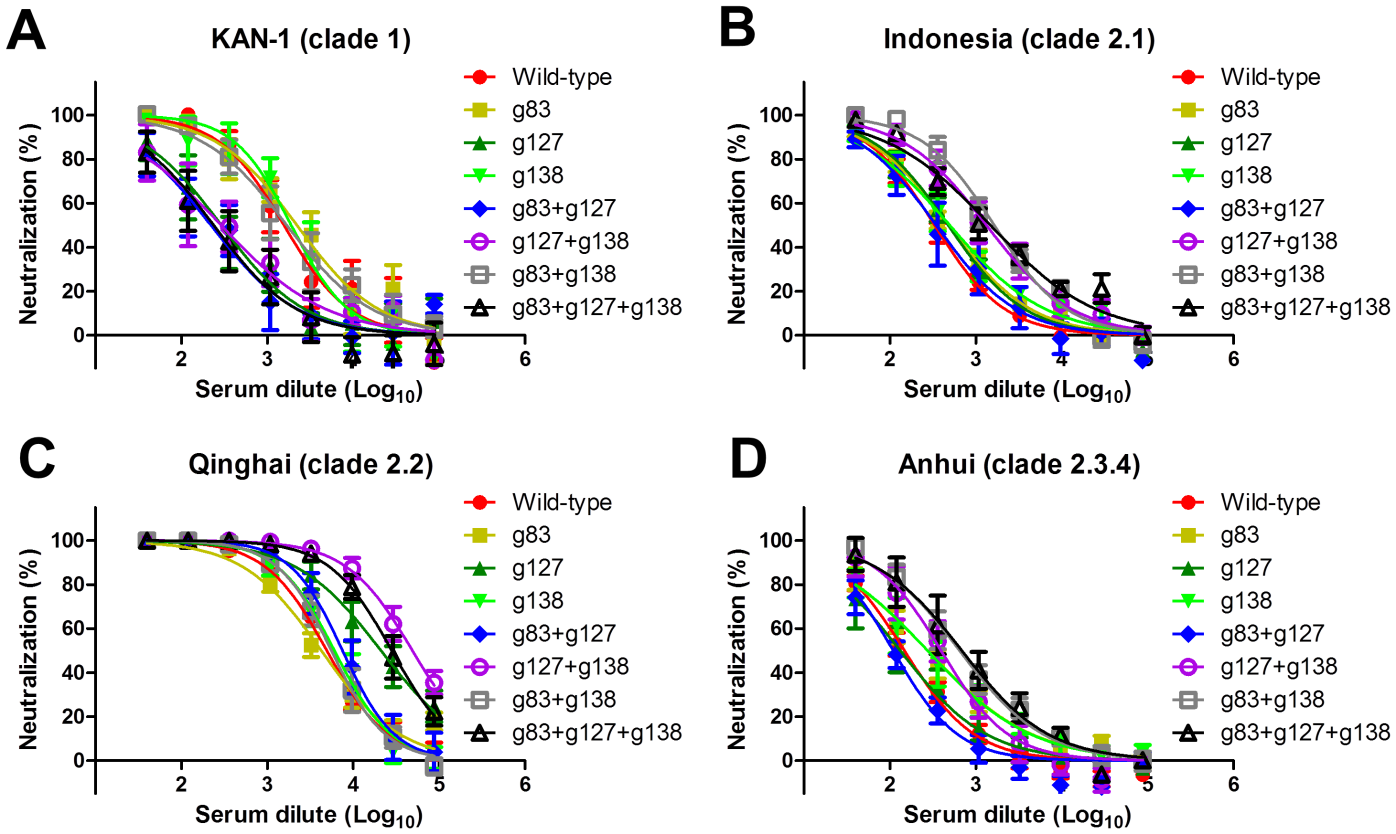
Neutralizing antibody titers elicited by glycan-masked H5HA mutant antigens. Serum dilution neutralization curves were obtained using H5pp containing HA from (A) KAN-1 (clade 1), (B) Indonesia (clade 2.1), (C) Qinghai (clade 2.2), or (D) Anhui (clade 2.3.4) strains.

**Figure 6:**
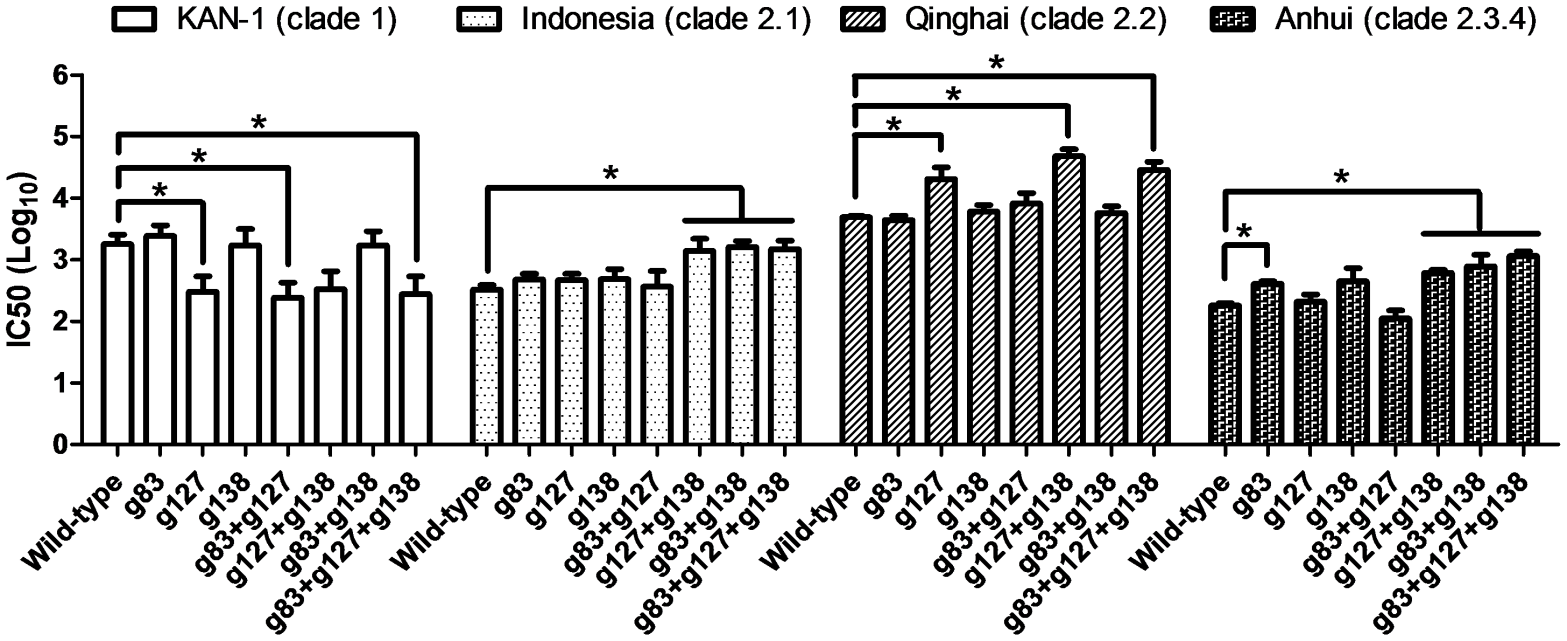
Neutralization titers are shown as IC50 values calculated from neutralization curves for H5pp containing HA from KAN-1 (clade 1), Indonesia (clade 2.1), Qinghai (clade 2.2), or Anhui (clade 2.3.4) strains.

We also measured neutralizing antibodies elicited by the immunizations of WT, single, double, and triple mutants of glycan-masked H5HA antigens against homologous and heterologous clades of H5N1 viruses. Serum-dilution neutralization curves for each immunization group were shown against H5pp of KAN-1, clade 1 (Figure 5A); Indonesia, clade 2.1 (Figure 5B); Qinghai, clade 2.2 (Figure 5C); and Anhui, clade 2.3.4 (Figure 5D). Corresponding IC50 values were calculated from neutralization curves to give half maximal (50%) inhibition for the dilution of the sera. The results indicate that glycan-masked g127, g83+g127 and g83+g127+g138 mutants had reduced IC50 values for the homologous KAN-1, clade 1 strain of H5pp (Figure 6). In contrast, the glycan-masked g127+g138, g83+g138 and g83+g127+g138 mutants had increased IC50 values for the Indonesia clade 2.1 H5pp (Figure 6). The glycan-masked g127, g127+g138 and g83+g127+g138 mutants had increased IC50 values for the Qinghai clade 2.2 H5pp (Figure 6). The glycan-masked g83, g138, g127+g138, g83+g138 and g83+g127+g138 mutants had higher IC50 values for the Anhui clade 2.3.4 H5pp (Figure 6). All together, the data indicate that the glycan-masked g127+g138 mutant elicited significantly broader HI and neutralizing antibody responses against heterologous H5N1 virus strains. However, these sera did not elicit significant titers of HI and neutralizing antibodies against H1N1pdm09, H3N2 and H7N9 viruses (data not shown).

### Mapping RBS-specific Antibodies Elicited by Glycan-masked H5HA Immunization

**Figure 7:**
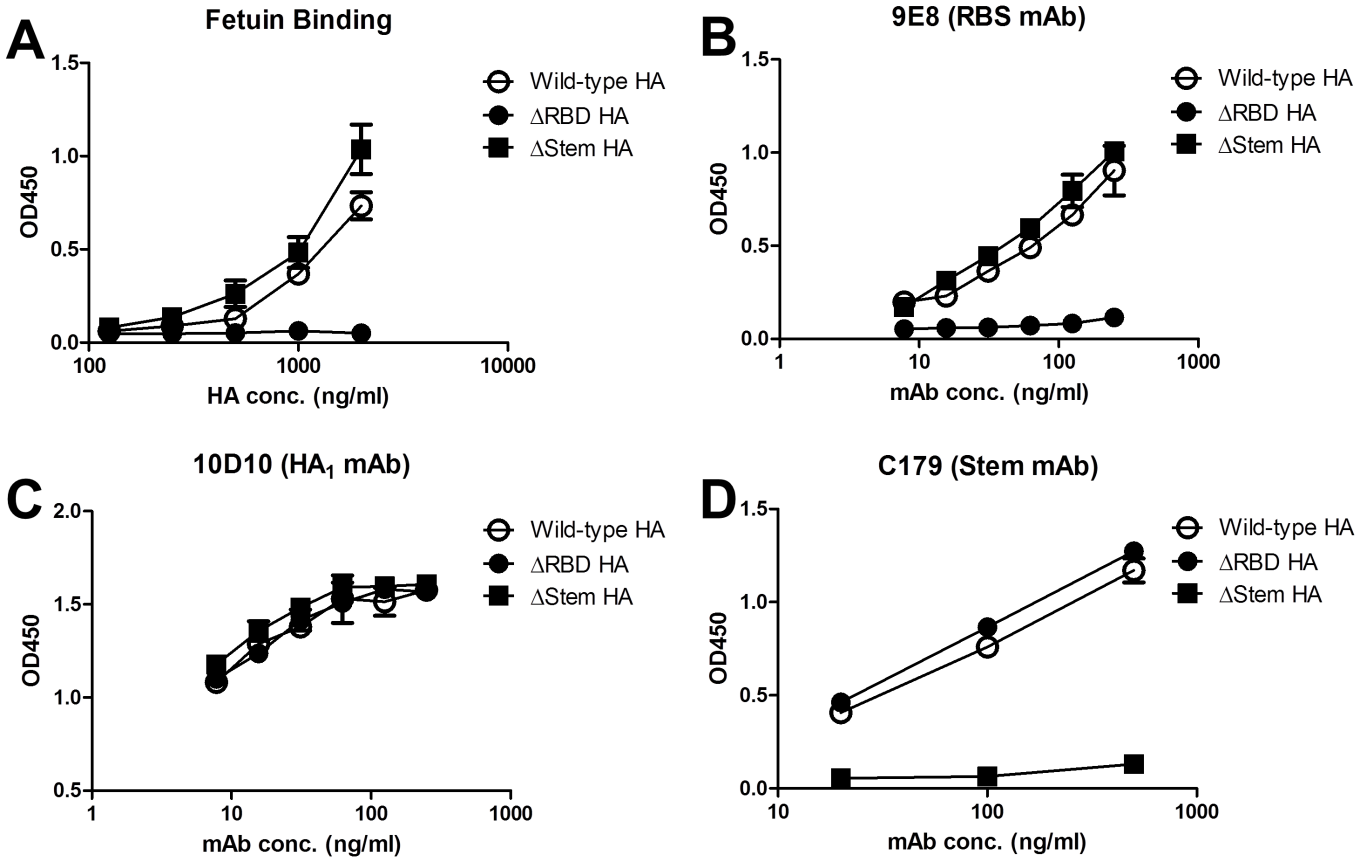
Identification of wild type-H5HA, ΔRBS-H5HA and ΔStem-H5HA proteins. (A) Serially diluted H5HA proteins (wild type, ΔRBS, ΔStem) were added to plates coated with fetuin and measured for ELISA binding. Different concentrations of (B) mAb 9E8 (targeted to the RBS 190 helix), (C) mAb 10D10 (targeted to the HA1 150 loop), and (D) mAb C179 (targeted to stem region) were reacted with each H5HA protein for ELISA binding.

**Figure 8:**
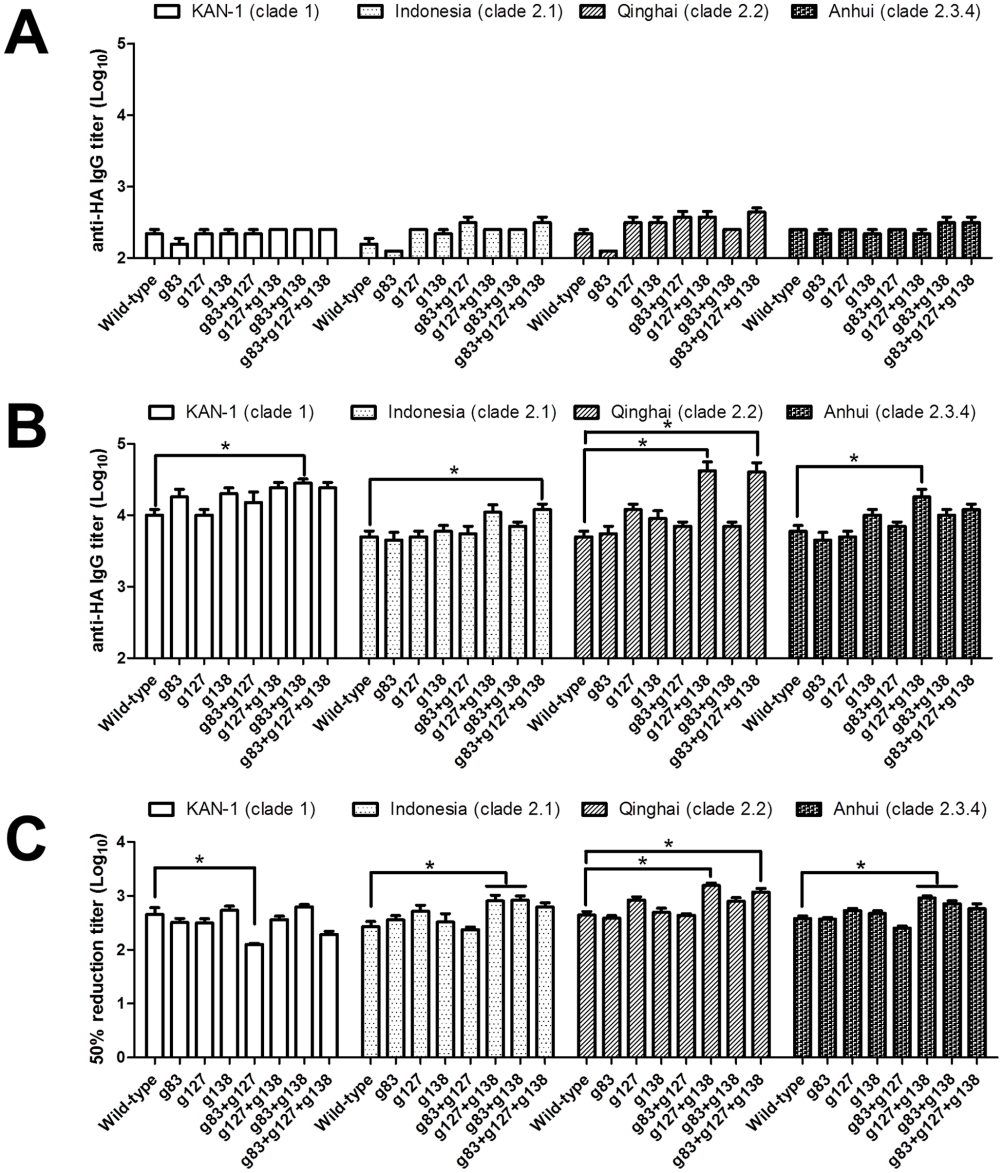
Mapping of RBS-specific antibodies elicited by glycan-masked H5HA mutants. Sera were pre-absorbed with (A) wild-type H5HA (KAN-1) protein or (B) ΔRBS-H5HA. ELISAs were performed to measure the HA-specific IgG titers of pre-absorbed sera against different HAs. (C) Pre-absorbed sera were also analyzed using fetuin-based serum inhibition assays to confirm RBS-specific antibody responses. Reduction titers (50%) of RBS-specific antibodies were measured as reduced fetuin binding to different HAs. Data represent geometric mean ± standard deviation. Results were analyzed using one-way ANOVAs and Tukey’s tests (*, statistical significance at p<0.05).

Since relatively conserved RBS represents a target for eliciting a broad spectrum of antibodies in contrast to other antigenic sites [22], we constructed a H5HA mutant protein containing an additional N-glycan at residue 186 on the RBS 190 helix (ΔRBS-H5HA) [23] to map RBS-specific antibody responses elicited by glycan-masked HA proteins. The E186N change in the RBS mutant (ΔRBS-H5HA) allowed for the introduction of an N-glycan into the RBS 190 helix, and I375N and a G377T changes in the stem mutant (ΔStem-H5HA) supported the introduction of an N-glycan into the mid-stem helix A. Mutant protein binding was confirmed using fetuin (Figure 7A), mAb 9E8 (targeted to the RBS 190 helix) (Figure 7B), mAb 10D10 (targeted to the HA1 150 loop) [24] (Figure 7C), and a stem-specific mAb C179 [25] (Figure 7D). Following antisera pre-absorption with the wild-type H5HA (KAN-1) protein, ELISA assays indicated relatively low levels of residual antibodies reacting with the wild-type H5HA proteins of the KAN-1, Indonesia, Qinghai, and Anhui strains (Figure 8A), suggesting that the antibodies induced by these single, double, and triple glycan-masked HA immunizations primarily reacted with the wild-type H5HA protein. For the RBS-specific IgG titers against the KAN-1 strain (with ΔRBS-H5HA used to remove non receptor site-directed antibodies), all of the glycan-masked mutant-generated sera had similar or higher values for the KAN-1 strain compared with sera raised against wild-type KAN-1 HA. For RBS-specific IgG titers against the heterologous strains, the glycan-masked g127+g138 mutant was significantly higher for the Qinghai and Anhui strains, and the glycan-masked g83+g127+g138 mutant was significantly higher for the Indonesia and Anhui strain, all compared to the wild-type H5HA (Figure 8B). Overall, the glycan-masked g127+g138 and g83+g127+g138 mutants were the more effective H5HA antigens in terms of inducing a broader range of RBS-specific IgG antibodies.

Fetuin-based serum inhibition assay was used to further confirm the RBS-specific antibody responses elicited by the glycan-masked H5HA immunizations. For the 50% reduction titers of RBS-specific antibodies, we used antisera to measure the reduction of fetuin binding to the recombinant H5HA proteins of the KAN-1, Indonesia, Qinghai, and Anhui strains. For reduction titers against the homologous strain, the glycan-masked g83+g127 mutant was significantly lower for the KAN-1 strain. For reduction titers against the heterologous strains, the glycan-masked g127+g138 and g83+g138 mutants were significantly higher for the Indonesia and Anhui strains, and the glycan-masked g127+g138 and g83+g127+g138 mutants were significantly higher for Qinghai strain compared to the wild-type H5HA (Figure 8C). In other words, the glycan-masked g127+g138 mutant elicited the highest levels of RBS-specific antibodies inhibiting the receptor binding of the three heterologous H5N1 virus clades.

### Mapping Stem-specific Antibodies Elicited by Glycan-masked HA Immunization

**Figure 9:**
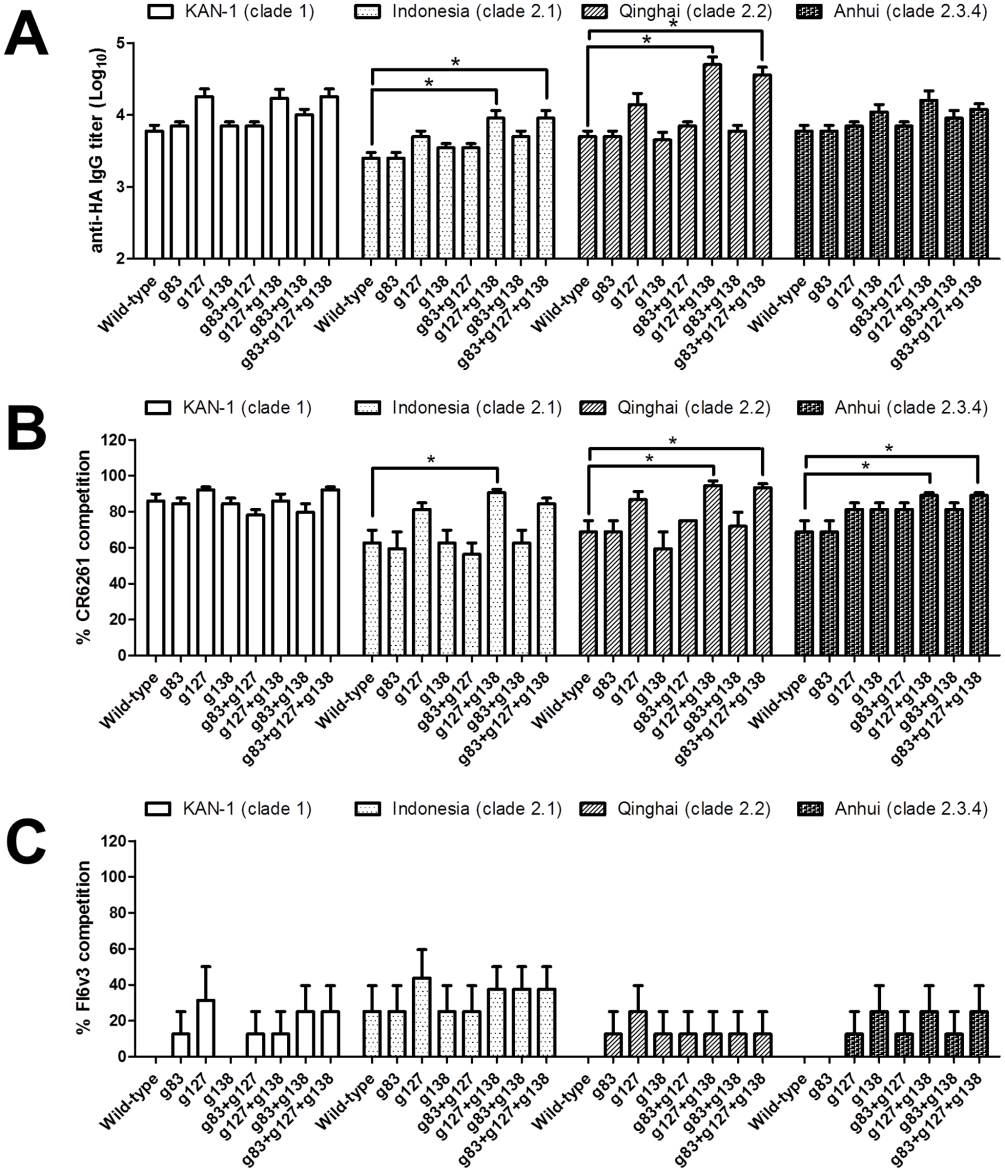
Mapping of stem-specific antibodies elicited by glycan-masked H5HA mutants. Sera were pre-absorbed with (A) ΔStem-H5HA. ELISAs were performed to measure HA-specific IgG titers of pre-absorbed sera against different HAs. Antibody competition assays were performed using (B) mAb CR6261 and (C) mAb FI6v3. Percentages of mAb competition to block binding between pre-absorbed sera and different H5HA proteins were calculated. Data represent geometric mean ± standard deviation. Results were analyzed using one-way ANOVAs and Tukey’s tests (*, statistical significance at p<0.05).

Since several stem-directed antibodies were found to neutralize viruses by binding to the highly conserved HA stem region that is essential for fusion [22], we constructed a H5HA mutant protein containing an additional N-glycan at residue 375 in the conserved stem region (ΔStem-H5HA) [26] to map stem-specific antibody responses elicited by glycan-masked HA proteins. Fetuin, mAb 9E8, mAb 10D10, and mAb C179 were used to confirm specific instances of binding. For stem-specific IgG titers against the homologous strain (using ΔStem-H5HA to remove non-specific antibodies), we observed that the g127, g127+g138 and g83+g127+g138 mutants were higher for the KAN-1 strain. For stem-specific IgG titers against heterologous strains, the g127+g138 and g83+g127+g138 mutants were higher for the Indonesia and Qinghai strains, and the g127+g138 mutant was higher for the Anhui strain compared to the wild-type H5HA (Figure 9A). According to these results, the g127+g138 mutant was the most effective H5HA protein for inducing a broad range of stem-specific antibodies.

To further investigate the bindings of different cross-reactive stem-specific antibodies, competition assays were performed using mAb CR6261 (capable of neutralizing the H1, H2, H5, H6, H8 and H9 subtypes) [27] and mAb FI6v3 (capable of neutralizing the H1, H3, H5 and H7 subtypes) [28]. The IgG titers of antisera pre-absorbed with ΔStem-H5HA were measured by ELISAs coated with different H5HA recombinant proteins blocked with mAbs, and percentages of blocked binding between pre-absorbed antisera and different H5HA proteins were calculated. Compared to the control antibody, mAb CR6261 significantly inhibited pre-absorbed antisera binding to the HA proteins of the KAN-1 (clade 1), Indonesia (clade 2.1), Qinghai (clade 2.2), and Anhui (clade 2.3.4) strains. For antibodies against the homologous strain, all glycan-masked mutants elicited similar levels of stem-specific antibodies competing with mAb CR6261 for the KAN-1 strain (Figure 9B). For antibodies against the heterologous strains, the g127+g138 mutant elicited higher levels of stem-specific antibodies competing with mAb CR6261 for the Indonesia strain (clade 2.1), and the g127+g138 and g83+g127+g138 mutants elicited higher levels of stem-specific antibodies competing with mAb CR6261 for the Qinghai (clade 2.2) and Anhui (clade 2.3.4) strains compared to the wild-type H5HA. Unlike the mAb CR6261 competition, the stem-specific antibody titers elicited by all of the glycan-masked mutants (including g127+g138 and g83+g127+g138) were not outcompeted by mAb FI6v3 (Figure 9C). The stem-specific antibodies elicited by the g127+g138 double mutant were CR6261-like, but not FI6v3-like.

### Cross-clade Protection in mice Following Live Virus Challenge

**Figure 10:**
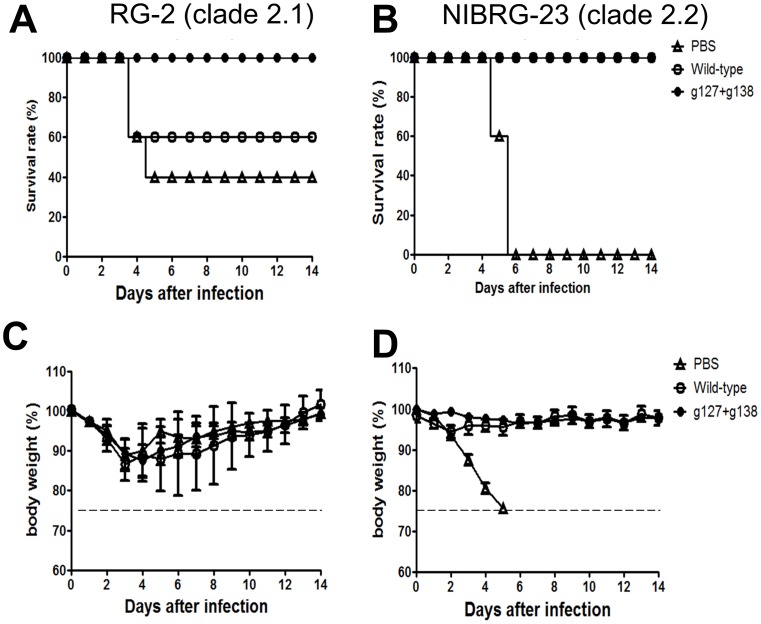
Protective immunity against influenza viruses challenges. The immunized mice were intranasally challenged with the reassortant RG2 (clade 2.1) or NIBRG23 (clade 2.2). After virus challenge, survival and body weight were recorded for 14 days. The body weight of each immunized group was presented as mean ± standard deviation. Over 25% body weight loss was regarded as an end-point.

To investigate the cross-clade protection elicited by the glycan-masked g127+g138 mutant, immunized mice were challenged with heterologous clades of H5N1 live viruses (RG-2 and NIBRG-23) to assess the protective immunities. For RG2 (clade 2.1) virus challenge, a complete protection was observed for immunization with the glycan-masked g127+g138 mutant as compared to 60% survival for the wild-type H5HA and 40% for the PBS control immunizations (Figure 10A). No significant differences in reduced body weight loss were found among these three groups (Figure 10C). For NIBRG-23 virus (clade 2.2) challenge, the glycan-masked g127+g138 mutant and the wild-type H5HA immunizations all elicited 100% protection as compared to 0% for the PBS immunization (Figure 10B). Again, no significant differences in reduced body weight loss were found between the wild type and the g127+g138 mutant immunizations as compared to 0% for the PBS immunization (Figure 10C).

## Discussion

Glycan masking to avoid antibody neutralization by sterically blocking antibody-epitope interaction is a strategy used by viruses such as influenza and HIV [29], [30]. H5N1 viruses have evolved into distinct antigenic clades and subclades, and uncertainty regarding which strain will be involved in an expected pandemic outbreak increases the stakes for making the correct selection for vaccine development [18]. In a previous study we reported that the glycan-masked g83 and g127 mutants elicited higher titers of cross-clade H5N1 neutralizing antibodies [19]. Additional evidence from this study points to the g138 mutant as also eliciting higher titers for the same antibodies. We found that the glycan-masked g127+g138 and g83+g127+g138 mutants elicited a significantly broader range of neutralizing antibody responses against heterologous H5N1 virus strains by increasing amounts of RBS-specific and stem-specific antibodies.

The present results indicate that the immunization strategy by priming with the adenovirus vector followed by a recombinant H5HA protein booster elicited approximately one-log increased HI titers and 0.3- to 0.4-log increased neutralizing antibody titers, as compared to our previous immunization strategy using two-dose DNA priming, followed by virus-like particles (VLPs) booster [19]. These results indicate that single-dose adenovirus vector outcompeted two-dose DNA vector in priming the immune responses by immunization. We also found that the booster with recombinant H5HA protein in PELC/CpG adjuvant was as effective as the booster with flagellin-adjuvanted VLPs as reported previously to improve anti-influenza immunity [31]. However, some discrepancy in the cross-neutralizing antibodies elicited by the glycan-masked g87 mutant using DNA-primed, VLP-boosting immunization [19] was not shown in the present studies using adenovirus vector-primed and recombinant H5HA protein boosting immunization. Whether two-dose DNA priming with the glycan masked g83 H5HA mutant may provide better cross reactivity for B cell priming and activation is still unclear.

According to the three-dimensional H5HA structures shown in Figure 1, residues 127 and 138 are located on the outer HA surface (close to the RBS 130 loop), and residue 83 is near the HA monomer interface. Further, residue 127 is located at the Sa site (H1), residue 83 at the E site (H3), and residue 138 at the A site (H3) [32]. All of the single, double, and triple mutants of the glycan-masked H5HA antigens at residues 83, 127 and 138 elicited similar or higher levels of cross-clade neutralizing antibodies. In contrast, the mutants containing g127 elicited lower neutralization titers against the homologous KAN-1 strain, whereas the mutants containing g83 or g138 did not (Figure 4, KAN-1). We also observed that the glycan-masked g127 and g83+g127 mutants had weaker bindings to epitopes on the 190 helix by mAb 9E8, but not to the epitopes on the 150 loop by mAb 10D10 (Fig. S1). According to one recent report, the Sa antigenic site in H1 masked by N-glycan on residue 144 (corresponding to residue 126 of H5HA, close to the 130 loop) elicited a broader polyclonal response capable of cross-neutralizing pH1N1 viruses; this is explained by the status of Sa as a major site for HI and neutralizing activities [33]. Accordingly, glycan-masked mutants containing g127 may block the KAN-1-specific immune-dominant region at the Sa site, resulting in reduced neutralization titers against the homologous KAN-1 strain.

Since H1, H3 and H5 HA RBS consists of a 130 loop, 190 helix, and 220 loop [24], we constructed a mutant H5HA (ΔRBS-H5HA) with an additional N-glycan on the 190 helix to determine RBS-specific antibodies, as previously described [23]. Results from absorption assays using ΔRBS-H5HA indicate that the glycan-masked g127+g138 and g83+g127+g138 mutants elicited increased cross-clade HI and neutralization titers, as well as higher levels of RBS-specific antibodies and antibodies inhibiting fetuin binding in the heterologous Indonesia (clade 2.1), Qinghai (clade 2.2) and Anhui (clade 2.3.4) strains (Figure 8). The unchanged levels of RBS-specific antibodies elicited by the g127 mutant indicate that the RBS-specific antibodies were not restricted to the Sa site. According to one recent study, relatively conserved RBS represents a target for eliciting a broad spectrum of antibodies in contrast to other antigenic sites [22]. We also noted a correlation between increased RBS-specific antibodies from the glycan-masked g127+g138 and g83+g127+g138 mutants and increased neutralizing antibody titers. There have been several reports indicating that RBS-targeting mAbs such as C05 and S139/1 neutralize both group 1 (H1, H2, H5, H6, H8 and H9) and group 2 (H3 and H7) subtypes [34]–[36], and that the RBS-targeting mAbs CH65, 1F1, and 5J8 neutralize divergent H1 (group 1) viruses [37]–[39]. However, increases in RBS-binding antibodies by the glycan-masked g127+g138 and g83+g127+g138 mutants were restricted to H5N1 viruses, not other subtypes of H1N1 and H3N2 viruses (data not shown).

Several HA stem-specific mAbs have been shown to cross-react with and neutralize several virus subtypes [28], [40]–[43]. This suggests that an engineered antigen eliciting stem-specific antibodies has the potential to serve as a universal vaccine. According to the mapping of stem-specific antibodies elicited by glycan-masked mutants (using ΔStem-H5HA), the g127+g138 and g83+g127+g138 mutants elicited the highest levels of stem-specific antibodies against the heterologous Indonesia (clade 2.1) and Qinghai (clade 2.2) strains (Figure 9A). Refocused stem-specific antibodies from the g127+g138 and g83+g127+g138 mutants overlapped with the broadly neutralizing epitopes of mAb CR6261 (Figure 9B) but not of mAb FI6v3 (Figure 9C). mAb CR6261 is known to neutralize most group 1 subtypes (i.e., H1, H2, H5, H6, H8 and H9) [27], [41], while mAb FI6v3 neutralizes group 1 (H1 and H5) and group 2 subtypes (H3 and H7) [28]. Both of these stem-specific antibodies have been described as neutralizing viruses by binding to the highly conserved stem region so as to prevent virus-cell fusion [22]. Although mAb CR6261 and mAb FI6v3 overlap on HA binding sites, they differ in terms of antibody-antigen interaction [28]. Several researchers have described stem-specific antibodies as having protective immunities even though they do not express neutralization activity [44]–[47]. The results indicated that the glycan-masked g127+g138 mutant not only elicited more CR6261-like stem-specific antibodies but also provided improved protective immunity against the heterologous RG-2 virus challenge (Figure 10A). The protective immunity of CR6261-like stem-specific antibodies elicited by glycan-masked g127+g138 and g83+g127+g138 mutants requires more detailed investigation by isolating individual B cell clones induced by the glycan masked mutant immunization.

## Materials and Methods

### Ethics Statement

The animal studies were conducted in accordance with guidelines established by the Laboratory Animal Center of National Tsing Hua University (NTHU). Animal use protocols were reviewed and approved by the NTHU Institutional Animal Care and Use Committee (approval no. 09931). Mouse challenge experiments were evaluated and approved by the Institutional Animal Care and Use Committee of Academia Sinica. Mice survived from immunization experiments were sacrificed using carbon dioxide (CO2) following ISCIII IACUC guidelines to ameliorate suffering. We also reported the data from mouse immunization studies in accordance with the ARRIVE guidelines.

### Recombinant H5HA Protein Construction and Purification

Soluble H5HA proteins were constructed using four HA cDNA sequences: A/Thailand/1(KAN-1)/2004 (KAN-1, clade 1), A/Indonesia/5/2005 (Indonesia, clade 2.1), A/bar-headed goose/Qinghai/1A/2005 (Qinghai, clade 2.2), and A/Anhui/1/2005 (Anhui, clade 2.3.4). The A/Thailand/1(KAN-1)/2004 HA gene was kindly provided by Prasert Auewarakul of Siriraj Hospital at Mahidol University, Thailand. The PQRERRRKKRG multibasic protease cleavage site between HA1 and HA2 was mutated to PQRETRG to prevent furin cleavage in cells. To obtain a trimeric H5HA protein, the C-terminus of the HA ectodomain was serially fused with a thrombin cleavage site, the GCN4-pII leucine zipper sequence, and a His-tag to facilitate protein purification. For large-scale production, Sf9 cells (Invitrogen) were grown in SF900-II serum-free medium (Invitrogen) at a density of 2×10^6^ cells/ml prior to infection with recombinant baculoviruses produced by the Bac-to-Bac expression system (Invitrogen). After 2 d post-infection, supernatants were collected for trimeric H5HA purification using nickel-chelated affinity chromatography (Tosoh). Trimeric H5HA protein expression was determined by SDS-PAGE and Western blots using polyclonal anti-H5HA antibodies (ab21297; Abcam).

A glycan-masked H5HA antigen design was introduced using site-directed mutations on residues 83 (^83^ANP^85^ replaced by ^83^NNT^85^ and named g83), 127 (^127^ASL^129^ replaced by ^127^NSS^129^ and named g127), and 138 (^138^QRK^140^ replaced by ^138^NGT^140^ and named g138) [19]. The RBS mutant H5HA protein (ΔRBS-H5HA) was constructed with the introduction of an N-glycan (E186N mutation) into the RBS 190 helix. The stem mutant H5HA protein (ΔStem-H5HA) was constructed with the introduction of an N-glycan (I375N and G377T mutations) in the mid-stem helix A. The H5HA mutants, also produced by the Bac-to-Bac expression system, were constructed as soluble trimeric forms. Purified H5HA mutants were confirmed by fetuin binding and antibody binding assays using mAbs 9E8, 10D10 and C179 (TaKaRa).

### Recombinant Adenovirus Vector Preparation

The ViraPower Adenoviral Expression System (Invitrogen) was used to create adenovirus vectors containing codon-optimized H5HA based on the A/Thailand/1(KAN-1)/2004 strain with a cleavage site mutation to retain uncleaved proteins. Briefly, a pENTR vector containing the H5HA gene was recombined (site-directed) with a pAd/CMV/V5-DEST vector using LR Clonase Enzyme Mix (Invitrogen). Following Pac I digestion, the recombined pAd/CMV/V5-DEST vector was transfected into HEK293A cells (Invitrogen) for adenovirus production. Recombinant adenoviruses encoding H5HA were produced 7 to 10 d post-transfection, with virus titers determined by plaque assays. H5HA proteins in cells infected with recombinant adenoviruses were confirmed by SDS-PAGE and Western blots using anti-H5HA antibodies.

### Mouse Immunization

Female BALB/c mice (6–8 weeks old; 5 mice per group) were intramuscularly primed with 10^8^ pfu of H5HA-encoding adenovirus vectors followed by 20 μg boosters of recombinant H5HA proteins coupled with PELC/CpG 3 weeks later [20], [21]. Sera were collected at week 5.

### Viral Challenge

Three weeks after the second immunization, the immunized mice were anesthetized and intranasally challenged with 10 LD_50_ of the reassortant H5N1 virus of RG2 (clade 2.1) or the reassortant H5N1 virus of NIBRG23 (clade 2.2) all in a final volume of 50 μl. PBS-immunized mice were used as a mock control. Mouse survival rates and weight losses were monitored daily for 14 d. According to IACUC guidelines, body weight loss over 25% was used as an end-point.

### ELISA Assays

Individual wells in 96-well plates were coated with recombinant HA proteins (0.2 μg/well) and blocked with 1% BSA. 2-fold serial dilutions of individual serum samples were incubated in each plate for 1 h and removed with 3 washes using PBS with 0.05% Tween-20. Goat anti-mouse IgG-conjugated HRP (Bethyl Laboratories, Inc.) was incubated in each well for 1 h followed by 3 additional washes. TMB substrate was incubated in each well for 15 min, followed by the addition of 2 N H_2_SO_4_ prior to readings at 450 nm absorbance. Endpoint titers were measured as the most dilute serum concentrations giving optical density readings >0.2 above a negative control [48].

### HI Assay

Sera were treated with receptor-destroying enzyme (Denka Seiken) for 18 h at 37°C followed by 56°C for 30 min to inactivate enzyme activity. Treated sera were two-fold serially diluted (starting from 1∶10) and incubated with 4 HA units of H5N1pp containing HA from KAN-1 (clade 1), Indonesia (clade 2.1), Qinghai (clade 2.2), or Anhui (clade 2.3.4) strains. Next, 0.5% turkey red blood cells were added and incubated for another 30 min at room temperature. HI titer was measured as the reciprocal of the highest dilution of sera which completely inhibiting hemagglutination.

### H5pp Neutralization Assay

Neutralizing antibodies were quantified as reduced luciferase expression levels following H5pp transduction in MDCK cells. 50 μl H5pp (50TCID_50_) was incubated with 50 μl of antisera (two-fold serial dilution, starting dilution 1∶40) for 1 h at 37°C followed by the addition of MDCK cells (1.5×10^4^ cells/well). At 2 d post-infection, cells were lysed with Glo Lysis Buffer (*Promega*). Luciferase activity was measured by the addition of neolite luciferase substrate (PerkinElmer). Neutralization titers (IC50) were measured as the serum dilution required to obtain a 50% reduction in RLU compared to control wells containing the virus only.

### Protein Absorption and Antibody Competition Assays

Protein absorption and antibody competition assays were performed as previously described [26]. For the protein absorption assays, mouse antisera was pre-absorbed with wild-type, ΔRBS, or ΔStem H5HA (40 μg/ml) for 1 h. Pre-absorbed antisera was used to measure IgG titers using ELISAs with the H5HA of the KAN-1, Indonesia, Qinghai, or Anhui strains. For the CR6261 antibody competition assays, ELISA plates coated with KAN-1, Indonesia, Qinghai, or Anhui H5HA were incubated with mAb CR6261 (10 μg/ml) for 1 h prior to the addition of antisera pre-absorbed with ΔStem H5HA; IgG titers were then measured by ELISA assays. Percentages of mAb CR6261 competition were calculated as (IgG titer with control antibody - IgG titer with CR6261)/IgG titer with control antibody ×100.

### Fetuin Binding and Fetuin Binding Inhibition Assays

Individual wells in 96-well plates were coated with 50 μg/ml of fetuin (Sigma), held overnight at 4°C, and blocked with 1% BSA in PBS buffer followed by three washes with 0.05% Tween 20/PBS buffer. Serially diluted soluble H5HA proteins were pre-mixed with HRP-conjugated anti-His tag antibodies (Bethyl Laboratories, Inc.) for 30 min, added to individual plates, and incubated for 60 min at room temperature. After three additional washes, H5HA binding was detected by ELISA assays (450 nm OD). For the fetuin binding inhibition assays, H5HA proteins (2 μg/ml) were pre-mixed with serially diluted antisera for 1 h prior to measuring fetuin binding activity as described above. Titers (50% reduction) were measured as the serum dilution required to obtain a 50% reduction in OD450 compared to control wells containing H5HA only.

### Statistical Analyses

All results were analyzed with one-way ANOVAs and Tukey’s tests using software GraphPad Prism v5.03, with *p*<0.05 indicating statistical significance. All experiments were performed at least two times.

## Supporting Information

Figure S1 **Glycan-masked H5HA proteins for HA1 binding epitopes.** ELISAs were performed to measure the binding levels of single, double and triple glycan-masked H5HA recombinant proteins to different concentrations of (A) mAb 9E8 (targeted to the HA1 RBS 190 helix), (B) mAb 10D10 (targeted to the HA1 150 loop).
(TIF)
