# Supplementary figures and images for: Glycan Masking of Hemagglutinin for Adenovirus Vector and Recombinant Protein Immunizations Elicits Broadly Neutralizing Antibodies against H5N1 Avian Influenza Viruses
Source: PLoS One. 2014 Mar 26;9(3):e92822. doi: 10.1371/journal.pone.0092822 (PMC3966833; doi:10.1371/journal.pone.0092822)

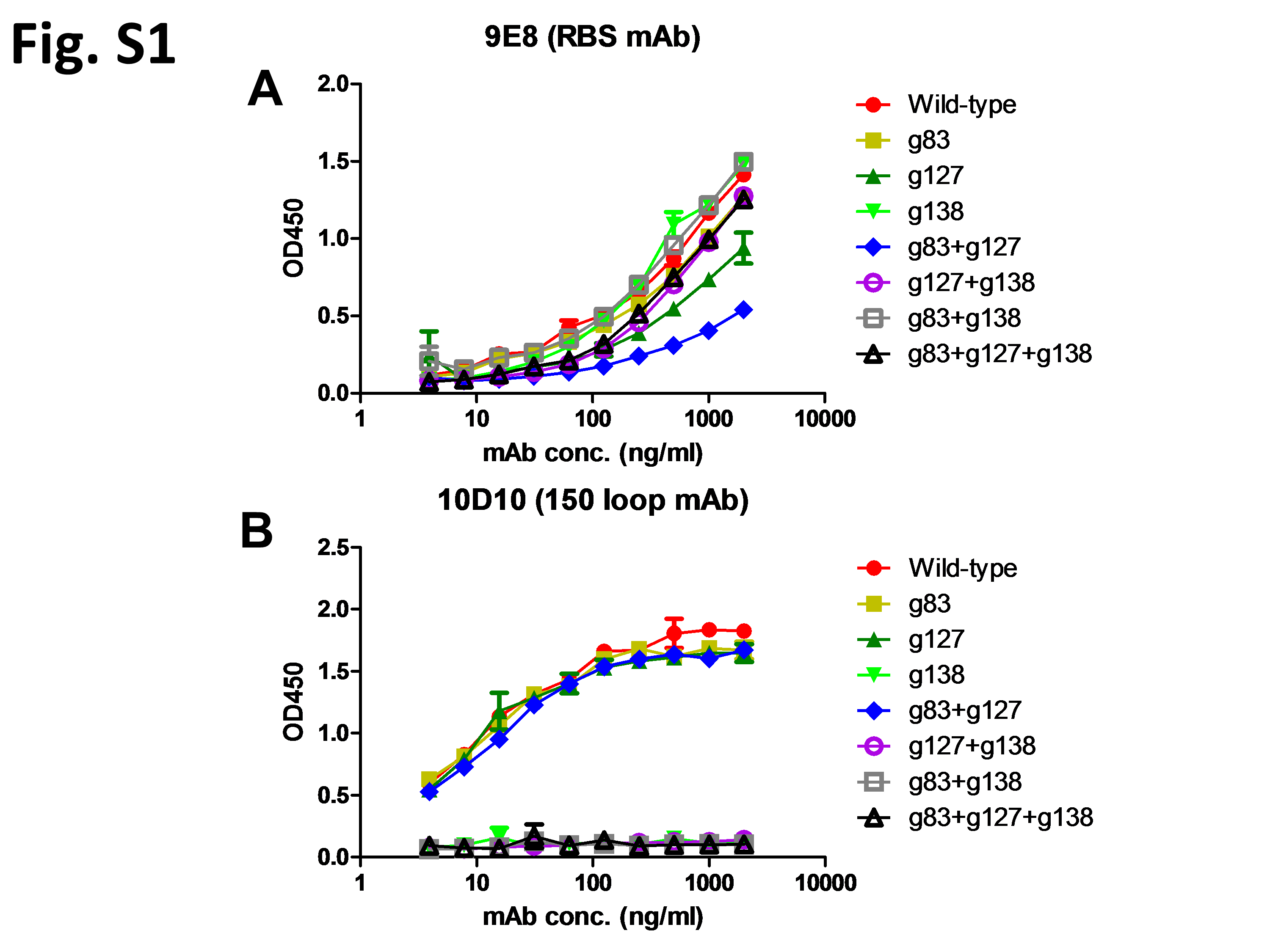

Supplement: Figure S1 — Glycan-masked H5HA proteins for HA1 binding epitopes. ELISAs were performed to measure the binding levels of single, double and triple glycan-masked H5HA recombinant proteins to different concentrations of (A) mAb 9E8 (targeted to the HA1 RBS 190 helix), (B) mAb 10D10 (targeted to the HA1 150 loop). (TIF) [file pone.0092822.s001.tif]
